# Letter to the editor regarding: ‘Electroacupuncture versus sham for insomnia in patients undergoing maintenance hemodialysis: study protocol for a pilot randomized controlled trial’

**DOI:** 10.1080/07853890.2025.2588912

**Published:** 2025-11-21

**Authors:** Zhuoyi Li, Luyao Fu, Ruijie Ma

**Affiliations:** ^a^ The Third School of Clinical Medicine (School of Rehabilitation Medicine), Zhejiang Chinese Medical University, Hangzhou, Zhejiang, China; ^b^ Department of Acupuncture and Moxibustion, Third Affiliated Hospital of Zhejiang Chinese Medical University, Hangzhou, Zhejiang, China

**Keywords:** Electroacupuncture, insomnia, hemodialysis, lettter

To the editor

We read with great interest the recently published protocol by Lu et al. titled ‘Electroacupuncture versus sham for insomnia in patients undergoing maintenance hemodialysis: study protocol for a pilot randomized controlled trial’ [1]. We commend the authors for their rigorous design and their efforts to address a prevalent but often overlooked clinical issue: insomnia in patients undergoing maintenance hemodialysis (MHD). However, we would like to offer several methodological reflections.

Firstly, the single-centre design and small sample size are notable limitations of this study. The data came from only one hospital. Acknowledging this, the authors may consider a multicentre follow-up trial to capture geographic and demographic diversity in future studies. In addition, although this pilot study was exploratory, effect-based sample-size emulation [2], even with wide confidence intervals, could have enhanced the interpretability and extensibility of the findings.

Secondly, the sham-controlled design could be further strengthened. Although the use of superficial needling at non-acupoints with disabled electrodes is a reasonable control, such interventions may still induce non-specific physiological effects. To better isolate the specific efficacy of electroacupuncture, additional measures such as a third no-treatment control group or the use of validated placebo needles (e.g. Streitberger needle) could be considered. Moreover, we recommend that the protocol and reporting be aligned with established standards, particularly STRICTA and ACURATE [3]. These frameworks emphasize the rationale for selecting both acupoints and control sites, detailed descriptions of needling and electroacupuncture parameters, the overall treatment regimen (timing relative to hemodialysis, and concomitant interventions). Importantly, ACURATE further requires specification of sham device characteristics, protocolized use and credibility assessments. Integrating these standards would substantially enhance reproducibility, transparency, and the credibility of the sham control in this trial.

Thirdly, the outcome indicators could be expanded to better reflect treatment efficacy. The primary outcome, defined as a 7-point reduction in ISI score, is clinically relevant but remains a subjective measure. Incorporating objective sleep measures [4] (e.g. actigraphy) or emphasizing sleep diary parameters in composite outcomes may provide a more comprehensive efficacy profile.

Furthermore, the statistical model of this protocol may be further refined. Although the use of mixed-effects models is appropriate, we also suggest clearly stating whether random effects like patient ID are used, and if covariates such as age, dialysis duration, and rescue medication are included in the analysis. To further control the risk of false positives, multiple testing of secondary outcomes should be corrected, preferably using the Benjamini–Hochberg [5] procedure for false discovery rate control.

Finally, Flexible scheduling may lead to differences in treatment dose. Adherence could be analysed as a continuous covariate or categorized by levels. Sensitivity analyses using this approach could help control for its effect.

In summary, this protocol reflects a commendable effort to bridge traditional medicine and modern clinical methodology. We look forward to the trial outcomes and hope our suggestions contribute to further refinement and broader applicability of this important work.

## Data Availability

Data sharing is not applicable to this article as no new data were created or analysed in this study.
